# Pulmonary Oxygen Toxicity in Navy Divers: A Crossover Study Using Exhaled Breath Analysis After a One-Hour Air or Oxygen Dive at Nine Meters of Sea Water

**DOI:** 10.3389/fphys.2019.00010

**Published:** 2019-01-25

**Authors:** Thijs T. Wingelaar, Pieter-Jan A. M. van Ooij, Paul Brinkman, Rob A. van Hulst

**Affiliations:** ^1^Diving Medical Center, Royal Netherlands Navy, Den Helder, Netherlands; ^2^Department of Anaesthesiology, Amsterdam UMC, University of Amsterdam, Amsterdam, Netherlands; ^3^Department of Pulmonology, Amsterdam UMC, University of Amsterdam, Amsterdam, Netherlands

**Keywords:** diving, pulmonary oxygen toxicity, exhaled breath analysis, gas chromatography–mass spectrometry, volatile organic components

## Abstract

**Introduction:** Exposure to hyperbaric hyperoxic conditions can lead to pulmonary oxygen toxicity. Although a decrease in vital capacity has long been the gold standard, newer diagnostic modalities may be more accurate. In pulmonary medicine, much research has focussed on volatile organic compounds (VOCs) associated with inflammation in exhaled breath. In previous small studies after hyperbaric hyperoxic exposure several methyl alkanes were identified. This study aims to identify which VOCs mark the development of pulmonary oxygen toxicity.

**Methods:** In this randomized crossover study, 12 divers of the Royal Netherlands Navy made two dives of one hour to 192.5 kPa (comparable to a depth of 9 msw) either with 100% oxygen or compressed air. At 30 min before the dive, and at 30 min and 1, 2, 3, and 4 h post-dive, exhaled breath was collected and followed by pulmonary function tests (PFT). Exhaled breath samples were analyzed using gas chromatography–mass spectrometry (GC–MS). After univariate tests and correlation of retention times, ion fragments could be identified using a standard reference database [National Institute of Standards and Technology (NIST)]. Using these fragments VOCs could be reconstructed, which were then tested longitudinally with analysis of variance.

**Results:** After GC–MS analysis, seven relevant VOCs (generally methyl alkanes) were identified. Decane and decanal showed a significant increase after an oxygen dive (*p* = 0.020 and *p* = 0.013, respectively). The combined intensity of all VOCs showed a significant increase after oxygen diving (*p* = 0.040), which was at its peak (+35%) 3 h post-dive. Diffusion capacity of nitric oxide and alveolar membrane capacity showed a significant reduction after both dives, whereas no other differences in PFT were significant.

**Discussion:** This study is the largest analysis of exhaled breath after in water oxygen dives to date and the first to longitudinally measure VOCs. The longitudinal setup showed an increase and subsequent decrease of exhaled components. The VOCs identified suggest that exposure to a one-hour dive with a partial pressure of oxygen of 192.5 kPa damages the phosphatidylcholine membrane in the alveoli, while the spirometry and diffusion capacity show little change. This suggests that exhaled breath analysis is a more accurate method to measure pulmonary oxygen toxicity.

## Introduction

In covert military dives of the Special Operating Forces most often pure oxygen is used in a closed-circuit rebreather system (Wingelaar et al., 2017). Exposure to higher than normal partial pressures of oxygen can lead to pulmonary oxygen toxicity (POT) (van Ooij et al., 2016). POT can be divided into an early exudative and a late proliferative phase (Winter and Smith, 1972; Bryan and Jenkinson, 1988). The first, caused by local inflammation with capillary and endothelial edema, leads to tracheobronchial irritation, coughing and retrosternal pain (Miller and Winter, 1981). This inflammatory process is reversible, while the irreversible proliferative phase develops when oxygen exposure is continued. The end state of this disease is proliferation of fibroblasts and type II alveolar cells, ultimately leading to fibrosis (Kapanci et al., 1972; Robinson et al., 1974).

The current safe limits of exposure to oxygen were determined by Clark and Lambertsen (1970). Bardin and Lambertsen (1970) established the concept of units of pulmonary toxicity dose (UPTD), based on a correlation between UPTD and a decrease in vital capacity (VC) after hyperbaric hyperoxic exposure in a dry chamber at rest. By consensus, a median decrease in VC of 2% (or 615 UPTD) was marked as the safe threshold for daily exposure. Even though the methodology is sound, the use of VC as the sole parameter to determine POT has its limitations (Harabin et al., 1987; van Ooij et al., 2012). The cardiovascular effects of immersion, hypothermia or physical exercise while diving on the human physiology are not accounted for in the UPTD model (Taylor and Morrison, 1990). Although attempts have been made to refine the UPTD model, basically the decrease in VC is still the main parameter (Arieli et al., 2002). Studies on the change of VC after “wet dives,” and research on new diagnostic modalities to determine POT, are limited (van Ooij et al., 2013, 2016). Therefore, the outdated UPTD model (almost 50 years after its conception) is still used as the gold standard to quantify POT.

Although newer diagnostic modalities in pulmonary function tests (PFT), such as diffusion capacity of nitric oxide (DL_NO_) and carbon monoxide (DL_CO_), can distinguish between air and oxygen dives, they show little promise to quantify POT (van Ooij et al., 2014a). Studies in pulmonary medicine are increasingly focussed on the detection of volatile organic compounds (VOCs). VOCs are metabolites of both physiological and pathological processes which can be detected in exhaled breath (Moser et al., 2005). Pattern recognition in VOCs has been helpful in diagnosing asthma, lung cancer and acute respiratory distress syndrome (Bos, 2018). Some small studies have confirmed that VOCs associated with inflammation, generally methyl alkanes, can be isolated after hyperoxic exposure, but the optimal time frame for measurement post-dive remains unknown (Morita et al., 1986; Lemaitre et al., 2002; van Ooij et al., 2014b). Although clinical manifestations develop faster at a higher partial pressure of oxygen (PO_2_), a PO_2_ as low as 27.5 kPa via nasal prongs at 2 L/min can induce oxidative stress (Phillips et al., 2003). Exhaled breath analysis seems a promising diagnostic modality for quantifying POT, but no longitudinal analysis of development of VOCs after hyperbaric hyperoxic exposure has been conducted.

Therefore, the present study aims to identify which VOCs mark the development of POT in divers, based on exhaled breath analysis. Measurements at several time points after an oxygen dive may provide more insight into the early onset of POT and identify the optimal period post-dive to determine POT.

## Materials and Methods

### Setting

This randomized crossover study was conducted at the Diving Medical Center, Royal Netherlands Navy (Den Helder). This study was carried out in accordance with the recommendations of the Ethics Committee of the University of Amsterdam and adhered to the principles of the Declaration of Helsinki with written consent from all subjects. The protocol was approved by the Medical Ethical Committee of the University of Amsterdam (Reference: 2017.183) and the Surgeon General of the Ministry of Defense. The study was registered at the Dutch Trial Register (ID: NTR6547).

Eligible for inclusion were healthy, non-smoking divers of the Royal Netherlands Navy. Eligible participants were fit to dive according to the European Diving Technology Committee standards, with the exception that PFT was assessed using the reference values of the Global Lung Function Initiative (Wendling and Nome, 2004; Wingelaar et al., 2018). Exclusion criteria were: recent respiratory tract infection, daily use of two or more alcoholic beverages, and/or the use of (over-the-counter) medication.

After being informed about the aims of the study, all participants gave consent. Informed consent was voluntary and could be retracted at any time without any consequences. According to privacy regulations, no study data were included in the medical file of the participants.

Participants were not exposed to hyperbaric conditions for at least 72 h prior to start of the study. During the study and the day before hyperbaric exposure, no strenuous physical exercise (including sports) was performed. To avoid affecting the exhaled breath profile, divers had to fast for 1 h before the first measurement and were only allowed to drink water. Between the third and fourth measurement food (bread and jelly) was provided and divers were encouraged to eat to prevent alteration of metabolism due to fasting (Fischer et al., 2015).

### Material and Measurements

Participants made two in-water dives of 60 min each to a pressure of 192.5 kPa (equal to 9 msw). The dives were performed in the wet compartment of the Medusa dive simulator (Haux Life Support, Germany). One dive was made while breathing air and the other while breathing 100% oxygen; the order of the dives was determined by lot. Whether the participant was breathing oxygen or air was unknown to both the divers and the researchers (double-blind), but was known to the technicians of the recompression facility due to safety regulations. Divers breathed via a surface supplied full-face breathing mask at a pressure of 192.5 kPa for 60 min. No physical activity was performed at depth in order to standardize conditions and to prevent central nervous system oxygen toxicity (Arieli et al., 2002).

Spirometry and DL_NO_ and DL_CO_ were measured with a Masterscreen PFT Pro (Carefusion, Netherlands) by qualified respiratory technicians according to the European Respiratory Society (ERS) Guidelines (Miller et al., 2005; Graham et al., 2017). Procedures for PFT are published elsewhere (van Ooij et al., 2014a). Baseline measurements were performed at least 24 h prior to or after participation in the study. To prevent forced expiratory maneuvres and exposure to carbon monoxide from affecting exhaled breath samples, PFT was performed once after all exhaled breath samples had been collected (i.e., after collection of the sixth sample: see next paragraph).

**FIGURE 1:**
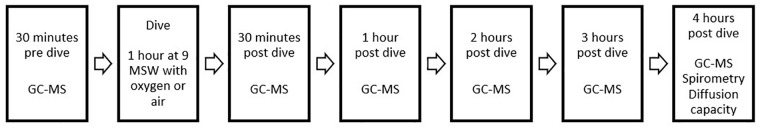
Overview of study design and data collection. Both study days were identical, the only difference being the type of exposure during the dive (either 100% oxygen or compressed air). Baseline measurements of spirometry and diffusion capacity were performed on a different day and are not shown here.

Exhaled breath samples were collected in accordance with ERS recommendations (Horvath et al., 2017). The diver breathed for 5 min through a disposable two-way non-rebreathing valve (Carefusion, Netherlands) combined with an inspiratory VOC filter (Honeywell, United States) to prevent contamination of exogenous particles. After 5 min, a single expiratory breath was collected in an empty uncoated aluminum balloon (Globos Nordic, Denmark). After collection, 500 mL of exhaled breath was pumped through a stainless steel tube filled with sorbent material (Tenax^TM^ GR 60/80, Camsco, United States) using a calibrated automatic air sampling pump (Gastec, Japan) at 250 mL/min, resulting in entrapment of VOCs. Pre-dive measurements were performed 30 min before hyperbaric exposure. Post-dive, the measurements were performed at 30 min and then at each full hour until 4 h post-dive (Figure 1).

Exhaled breath samples were analyzed using gas chromatography–mass spectrometry (GC–MS) based on standardized procedures (Fens et al., 2009). In short, the tubes were heated to 250°C for 15 min with a flow of 30 mL/min using a thermal desorption unit (Markes, United States), where VOCs were captured in a cold trap at 10°C. Then, the cold trap was rapidly heated to 300°C for 1 min, after which molecules were splitless injected in a 30 m gas chromatography column with a diameter of 0.25 mm at 1.2 mL/min (Restek, United States). Molecules were ionized using electron ionization at 70 eV. Fragments were detected using a quadrupole mass spectrometer (GCMS-GP2010, Shimadzu, Japan) with a scan range of 37–300 Da. Ion fragments were used for statistical analysis. The predictive fragment ions were manually checked in the raw chromatograms and the corresponding metabolites were tentatively identified based on the National Institute of Standards and Technology (NIST) library matching, using the OpenChrom software package (Wenig and Odermatt, 2010). Metabolites were considered identified if the first five hits in the library were the same compound and all matching factors were above 90%.

### Statistical Analysis

To our best knowledge, analysis of exhaled breath after hyperbaric hyperoxic exposure has only been reported in two small studies (Lemaitre et al., 2002; van Ooij et al., 2014b). However, whether this approach indeed yields results, and to what extent there is an effect on exhaled VOCs, remains unknown; this complicated calculation of the sample size. Since the above-mentioned studies included 7 and 10 participants, respectively, we decided that 12 participants would be sufficient to collect sufficient data.

After GC–MS analysis, an ion fragment peak table was generated, with de-noising, alignment and peak detection (signal-to-noise ratio 1:100) (Smith et al., 2006). A combined-batches algorithm was utilized to correct for possible batch effects (Johnson et al., 2007). Subsequently, data were tested (both univariately and paired) using Wilcoxon rank sum tests (i.e., two different time points with the same breathing gas, or the same time point with different breathing gases) to identify potentially relevant ion fragments. Then, ion fragments with retention times (±2 s) that correlated 0.98 or more were selected. From this selection of ion fragments/retention times compounds could be identified. The means (intensity of the GM-MS signal) of the compounds were longitudinally tested using a two-way analysis of variance (ANOVA) with correction for participant and test day, to detect differences between oxygen and compressed air over time. Similar to earlier studies, the intensities were also combined to create a “breathprint” of POT and tested separately (Phillips et al., 1999). The PFT data were univariately analyzed using Shapiro–Wilk and paired *t*-tests.

All statistical analyses were performed using the R software package (version 3.5.1, R Foundation for Statistical Computing, Austria), including the surrogate variable analysis (SVA version 3.7), Methods for the Behavioral, Educational, and Social Sciences (MBESS version 4.4.3) and Combined Batches (ComBat version 3.28.0) packages. A *p*-value of <0.05 was considered statistically significant.

## Results

Included in this study were 12 male, certified Navy divers (aged 36.1 ± 10.3 years; height 183.0 ± 6.2 cm; weight 90.5 ± 7.3 kg). All dives were without incidents. Directly post-dive the participants were asked to guess whether they had made the dive with air or oxygen: of the 24 guesses, 14 (58%) were correct. All PFT values were normally distributed and within the normal limits of the Global Lung Initiative (Table 1). Of all measured parameters, comparison of the mean data showed no significant differences between air and oxygen dives (paired *t*-tests). Compared with baseline, there was a significant decrease in DL_NO_ after both the air and oxygen dives, i.e., -2.19 mmol min^-1^ kPa^-1^ (*p* = 0.026) and -2.36 mmol min^-1^ kPa^-1^ (*p* = 0.003), respectively. Also, compared with baseline, 4-h after the oxygen dive the alveolar volume (V_A_) showed a slight decrease (-0.08 L, *p* = -0.025) whereas the decrease after the air dive was not significant (*p* = 0.12). Lastly, after both air and oxygen dives, there was a decrease in the alveolar membrane capacity (D_M_), i.e., 1.05 mmol min^-1^ kPa^-1^ (*p* = 0.043) and 1.17 mmol min^-1^ kPa^-1^ (*p* = 0.003), respectively.

**Table 1:** Data on pulmonary function tests at baseline and at four hours post-baseline for air and oxygen diving (*n* = 12 divers).

	Baseline	Air diving (4 h later)	Oxygen diving (4 h later)
FVC (L)	6.48 (0.66)	6.47 (0.75)	6.40 (0.74)
FEV_1_ (L)	5.00 (0.59)	4.98 (0.66)	4.92 (0.60)
FEV_1_/FVC	0.77 (0.08)	0.77 (0.08)	0.77 (0.08)
DL_CO_ (mmol min^-1^ kPa^-1^)	12.33 (1.44)	11.78 (1.53)	12.05 (1.55)
DL_NO_ (mmol min^-1^ kPa^-1^)	53.96 (5.67)	51.77 (6.15)^∗^	51.60 (6.25)†
DL_NO_/DL_CO_	4.46 (0.24)	4.49 (0.24)	4.43 (0.20)
V_A(SB)_ (L)	7.34 (0.82)	7.23 (0.82)	7.26 (0.85)^∗^
D_M_ (mmol min^-1^ kPa^-1^)	27.39 (2.86)	26.34 (3.21)^∗^	26.22 (3.19)†
V_C_ (mL)	96.46 (13.35)	90.95 (13.40)	95.58 (14.14)


*Data are mean (standard deviation). No significant differences were found when comparing oxygen and air dives. Significant difference versus baseline: ^∗^*p* ≤ 0.05 and ^†^*p* ≤ 0.01. FVC: Forced Vital Capacity, FEV_*1*_: Forced Expiratory Volume in one second, DL_*CO*_: diffusion capacity of carbon monoxide, DL_*NO*_: diffusion capacity of nitric oxide, V_*A (SB)*_: alveolar volume in a single breath, D_*M*_: alveolar membrane capacity, V_*C*_: capillary volume*.

Of the 144 GC–MS samples, 139 could be fully analyzed. One sample was contaminated and gave too much noise to analyze, two samples gave no signal (possibly a faulty connection during sampling), one sample was missing, and one technical malfunction in the mass spectrometer made analysis impossible.

**FIGURE 2:**
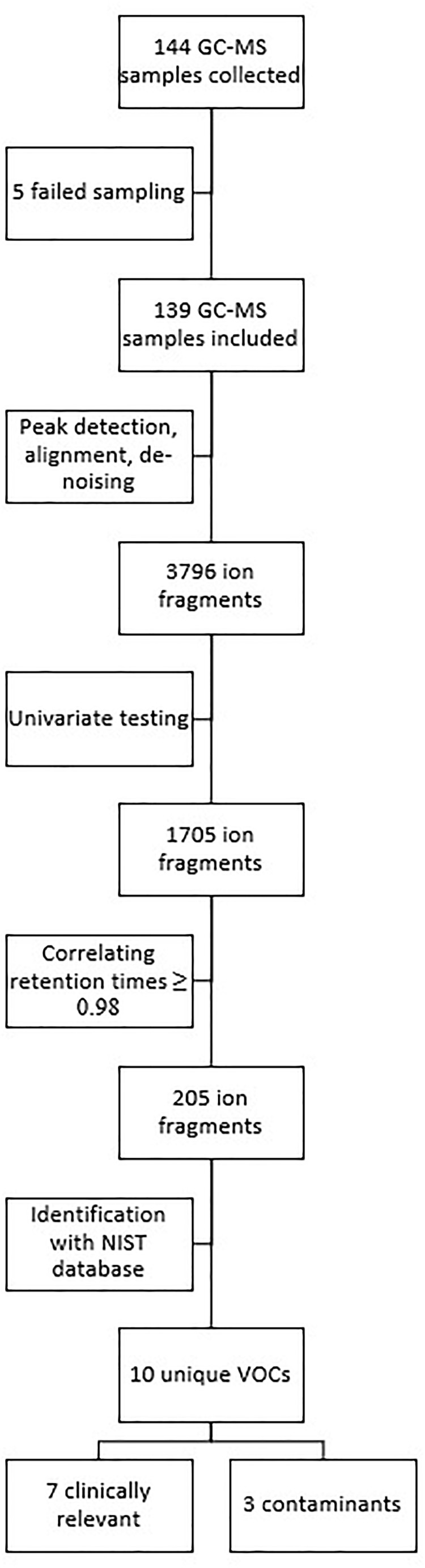
Overview of data and statistical analysis.

Analysis of the 139 samples led to the identification of 3796 different ion fragments, of which 1705 were significant (*p* ≤ 0.05) when tested univariately. Of those 1705 ion fragments, 205 had a retention time (±2 s) that showed a correlation of ≥0.98. When grouping these fragments using the Standard Reference Database (NIST), 10 unique VOCs were identified (Figure 2).

**FIGURE 3:**
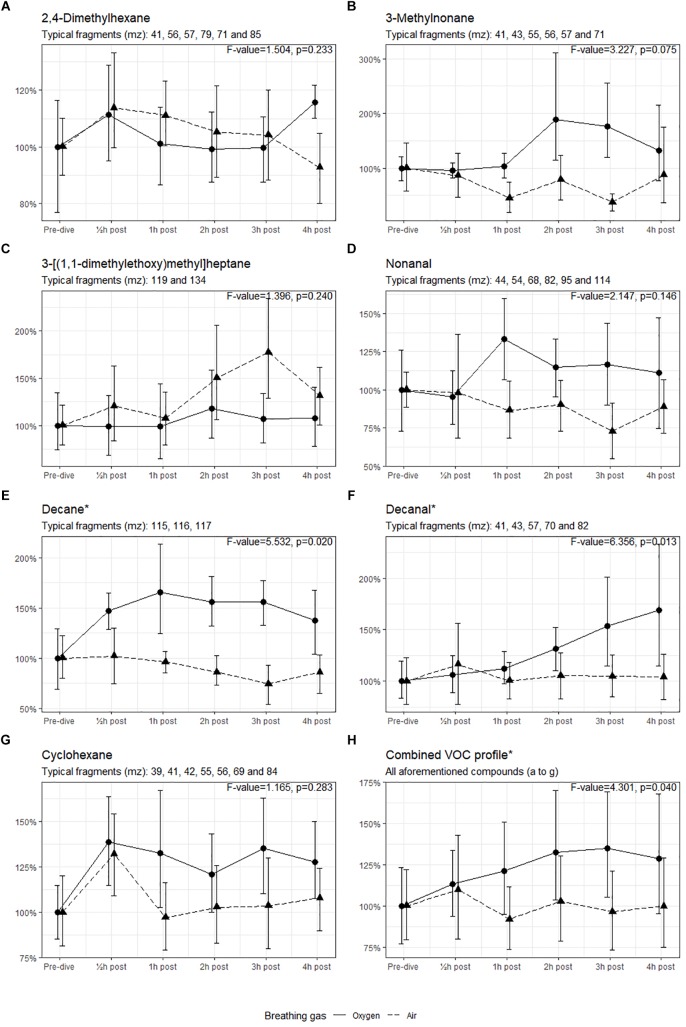
Intensity of the identified VOCs, with 95% CI. ^∗^Significant difference in mean values between oxygen and air dives. Results of the ANOVA are shown in the graph of each compound.

Seven compounds (generally, methyl alkanes) were identified as being endogenous from origin and thus relevant for analysis; their intensity over time is shown in Figure 3. The three remaining VOCs (hexamethylcyclotrisiloxane, butyl acetate and 1,2-dichloropropane) are generally considered to be contaminants and are not shown (D’ Angelo, 2011; National Center for Biotechnology Information, 2018).

The means of the VOCs in the oxygen group and the air group were tested using ANOVA (Figure 3). The intensities of decane and decanal were significantly higher in the oxygen group than in the air group (*p* < 0.05). When the intensities of all compounds were combined, the means in the oxygen group were higher (*p* = 0.040) with a 35% increase in intensity 3 h post-dive (mean 1.35, 95% CI 1.05–1.70) versus a 4% decrease at the same interval post-dive in the air group (mean 0.96, 95% CI 0.73–1.21).

## Discussion

To our knowledge this is the first study to longitudinally collect data on VOCs after hyperbaric hyperoxic exposure. Seven compounds were identified, of which decane and decanal were significantly increased after 60 min of oxygen diving to a depth of 9 msw. Combining these seven components revealed a significant increase after oxygen diving, which was at its peak 3 h post-dive. Additionally, there was no significant difference in the PFT values (whether spirometry or diffusion capacity) between air and oxygen diving. Apart from the decrease in DL_NO_ and D_M_ after both dives, the remaining PFT parameters (especially VC) remained almost unchanged. This suggests that VOCs are a more sensitive marker of the onset of POT than PFT.

Although a decrease in VC is at the core of the UPTD concept, its value has been questioned (Hruby and Butler, 1975; Shykoff, 2005; van Ooij et al., 2016; Wingelaar et al., 2017). For instance, after a dive of 60 min at a PO_2_ of 192.5 kPa (142 UPTD) the VC was expected to decrease by 0.15% (Bardin and Lambertsen, 1970; Arieli et al., 2002). However, in our population, it remained almost unchanged, with a non-significant decrease in VC of only 0.08 L (0.012%) after oxygen diving. Irrespective of our findings, it is debatable whether a 0.15% decrease in VC can be detected with PFT, as an intrapersonal variation of 5% is considered to be physiological (van Ooij et al., 2013). In our opinion, this illustrates the difficulty in utilizing a decrease in VC to determine POT. Both DL_NO_ and D_M_ were reduced after both dives, which can be attributed to alveolar inflammation with edema. However, it is unknown whether this is the result of immersion due to diving, or of (mild) elevated PO_2_ causing POT; these differences were not significant when comparing air and oxygen dives. This result is in contrast to earlier findings, despite that our study population and methodology were similar (van Ooij et al., 2014a). This might be attributed to the interpersonal variation of DL_CO_ and DL_NO_ (Murias and Zavorsky, 2007; Graham et al., 2017). The approximate 100 mL (± 1.5%) decrease in V_A_ was similar after both dives; however, after the oxygen dive this was significant with a *p*-value of slightly less than 0.05 and after the air dive was non-significant with a *p*-value slightly greater than 0.05. In view of the PFT results, it seems that none of the tested parameters was sufficiently sensitive to detect (early) POT.

This study confirms earlier findings on methyl alkanes after oxygen diving (van Ooij et al., 2014b). As the present 1-h dive to 9 msw accounts for 142 UPTD (i.e., less than 25% of the daily allowed UPTD), this also confirms that dives with relatively “low oxidative stress” alter exhaled breath profiles. Methyl alkanes are the result of lipid peroxidation of cell membranes due to hyperoxia (Phillips et al., 2003; van Ooij et al., 2014b). These compounds have also been found in several lung diseases (Phillips et al., 1999; Buszewski et al., 2007). Although GC–MS is the gold standard to identify exhaled compounds, this method is associated with some uncertainty. For instance, 2,4-dimethylhexane could also be 2,4-dimethylheptane or octane, as these molecules are highly similar in mass (Dalton) and mass-charge ratio and are difficult to distinguish from each other in GC–MS. Octane is also present in the adult respiratory distress syndrome (Bos et al., 2014b). Despite that we cannot be certain which compound has been detected (or perhaps a mixture of all three), all these compounds can originate from the oleate tail of the phosphatidylcholine membrane. More specifically, the distal part after the double bond, which is commonly found in the lung and as VOCs in pulmonary disease (Chen et al., 2018). Whether sustained oxidative damage to phosphatidylcholine leads to a reduction of VC is presently unknown, and therefore it may be difficult to link these results to the existing UPTD-concept or how these results could lead to an alternative to UPTD.

In the present study, in both air and oxygen dives 2,4-dimethylhexane emission was increased 30 min post-dive, but is notably more present at 4-h post oxygen dive (Figure 3A). This probably indicates that air dives at 9 msw (with a PO_2_ of 39.8 kPa) also induce POT, but not as severe as an oxygen dive with a PO_2_ of 192.5 kPa. This was also demonstrated by Phillips et al. (2003) after breathing 28% oxygen at 2.0 L/min in normobaric conditions. The increased intensity of 3-methylnonane (Figure 3B) after the oxygen dive has not previously been described, but this is also a methyl alkane. It seems feasible that this is due to a similar pathogenesis as in 2,4-dimethylhexane but, in this case, originating from the proximal part before the double bond in the oleate tail of phosphatidylcholine. Nonanal has been linked to pulmonary disease, such as lung cancer (Phillips et al., 1999; Yu et al., 2018). A similar pattern emerges for decane and decanal after oxygen dives, which can also originate from the same source as 3-methylnonane; both these compounds are associated with asthma and lung cancer (Caldeira et al., 2012; Yu et al., 2018). We cannot fully explain the non-significant higher intensity of 3-[(1,1-dimethylethoxy)methyl]heptane after the air dives (Figure 3C). Although there is a remarkable peak 3 h post-dive, this decreases rapidly thereafter; this has not been described earlier. This compound is similar to the 2,4-dimethylhexane and could originate from the same source. The higher emission of cyclohexane after an oxygen dive could be associated with inflammation; in pulmonary medicine this compound is associated with lung disease (e.g., mesothelioma) but is also found in intubated ICU patients (Bos et al., 2014a; Lamote et al., 2017). Although we know the natural variation of exhaled (methyl) alkanes, many of these compounds have been independently identified in several studies (Pereira et al., 2015). It seems likely that our results indicate that pulmonary damage occurs under hyperbaric hyperoxic conditions.

The longitudinal setup of our experiment allowed to measure VOCs over time. While only decane and decanal showed a significant increase when tested with ANOVA, most of the compounds follow a similar pattern after oxygen and air diving. This is best illustrated in Figure 3H, which shows a significant difference between the dives. In both cases there is an increased emission of VOCs 30 min post-dive, which normalizes shortly thereafter in the case of air diving. In oxygen diving this continues to increase until 3 h post-dive and then starts to decline. We remain uncertain about this observation, as no measurements were made beyond the 4 h post-dive. However, it is unlikely that a delayed reaction after air diving will occur ≥4 h post-dive.

After checking the correlation between the identified compounds, the highest R square was 0.332 (between decanal and nonanal). This might be attributed to different stages in the inflammation cascade and not only to direct lipoperoxidation due to hyperoxia. Further research is needed to unravel the hyperoxic inflammation cascade. Since the present study only included one dive profile (1 h at 9 msw), it is likely that different VOCs, or similar VOCs but at different intervals post-dive, will be found in future studies that use different dive profiles. For instance, it is unknown if POT occurs during the dive, or starts to develop after emerging. Based on the current data, future research on POT and VOCs should focus on the period 2–4 h post-dive; however, this needs to be confirmed using other dive profiles (i.e., at different diving time limits and different depths, and/or with different breathing gas mixtures) to exclude factors such as depth and PO_2_ in this response.

We identified three VOCs which are generally marked as contaminants. Hexamethylcyclotrisiloxane is commonly found in GC–MS analysis and is the result of column or septum bleed (D’ Angelo, 2011). Since silicon is not a part of human biochemistry, this was excluded from our analysis. 1,2-Dichloropropane was also found in both air and oxygen dives and probably originated from the coating on the inside of the hose used to supply the breathing gas. Lastly, butyl acetate was identified at several moments post-meal. When analyzed, this proved to be a food additive commonly used as a flavoring agent; this is the result of the diet we gave our divers (bread and jelly) (National Center for Biotechnology Information, 2018). Although this influenced our data, total fasting for 6 h would probably have had more impact on our results.

### Strength and Limitations

The double-blind crossover design of this study provides methodological strength, while measurements after in-water dives (in contrast to dry dives) give practical relevance to oxygen diving. To our knowledge this is the largest study on exhaled breath analysis in divers and the first to include multiple measurements post-dive. This allowed to detect potentially early inflammation, which could have decreased when measured at a later time point.

Some limitations also need to be addressed. Firstly, we did not include systemic biomarkers of oxidative stress (e.g., benzoate hydroxylation in urine or malondialdehyde in blood) as was done in similar studies (Loiseaux-Meunier et al., 2001; Gronow et al., 2005). Systemic biomarkers could have given additional proof of oxidative stress. However, systemic markers might originate from organ systems other than the lung and are, therefore, not entirely specific (Frijhoff et al., 2015; Valacchi et al., 2018). Additionally, in an earlier study with a similar oxygen exposure, malondialdehyde levels were not elevated, indicating low systemic oxidative stress (van Ooij et al., 2014b). We think that our results are organ specific and generate sufficient evidence for oxidative damage of alveolar membranes. Secondly, our population consisted of only healthy male, non-smoking Navy divers, which led to selection bias. Although this group is similar to our target population, our results cannot be extrapolated to either female divers or to less physically healthy divers. Lastly, with only 12 participants, the study may have been underpowered to detect a decrease of 0.15% in VC using PFT. While that may be the case, the question remains whether a decrease of such small magnitude is clinically relevant. This is in contrast to the significant results when testing decane, decanal and the combined profile of compounds, the latter with a power of 94.1%. We think this is an additional argument that VOCs are more accurate to detect the onset of POT than PFT. Nevertheless, this study reproduces the results of an earlier study in identifying methyl alkanes as potential markers of POT, despite the fact that our participants and methodology differed from that earlier study (van Ooij et al., 2014b). This seems to indicate that our results are not subject to bias.

## Conclusion

This study is the largest analysis of exhaled breath after submerged hyperbaric hyperoxic conditions (i.e., in-water oxygen dives) to date and the first to longitudinally measure VOCs. Seven VOCs could be identified; of these, decane and decanal were significantly increased after an oxygen dive for 60 min to 9 msw compared to an air dive. When combining all identified VOCs, the highest peak of VOCs in exhaled breath was at 3 h post-dive. After both dives, DL_NO_ and D_M_ showed a significant decrease, whereas no other differences between the remaining PFT parameters were significant. Although these results suggest that exhaled breath analysis is a more accurate method to measure early POT, they need to be confirmed when using other dive profiles and different compositions of breathing gas.

## Author Contributions

TW conceptualized, designed, and performed the experiments, and involved in statistical analysis, drafting, and revision of the manuscript. P-JO and RH conceptualized and designed the experiments, and involved in drafting and revision of the manuscript. PB designed and performed the experiments, and involved in GC–MS and statistical analysis, drafting, and revision of the manuscript.

## Conflict of Interest Statement

The authors declare that the research was conducted in the absence of any commercial or financial relationships that could be construed as a potential conflict of interest.
